# Binding peptide generation for MHC Class I proteins with deep reinforcement learning

**DOI:** 10.1093/bioinformatics/btad055

**Published:** 2023-01-24

**Authors:** Ziqi Chen, Baoyi Zhang, Hongyu Guo, Prashant Emani, Trevor Clancy, Chongming Jiang, Mark Gerstein, Xia Ning, Chao Cheng, Martin Renqiang Min

**Affiliations:** Machine Learning Department, NEC Labs America, Princeton, NJ 08540, USA; Computer Science and Engineering Department, The Ohio State University, Columbus, OH 43210, USA; Chemical and Biomolecular Engineering Department, Rice University, Houston, TX 77005, USA; Digital Technologies Research Centre, National Research Council Canada, Ottawa, ON K1A 0R6, Canada; School of Electrical Engineering and Computer Science, University of Ottawa, Ottawa, ON K1N 6N5, Canada; School of Medicine, Yale University, New Haven, CT 06520, USA; NEC OncoImmunity AS, Oslo Cancer Cluster, Oslo 0379, Norway; Department of Medicine, Baylor College of Medicine, Houston, TX 06520, USA; School of Medicine, Yale University, New Haven, CT 06520, USA; Computer Science and Engineering Department, The Ohio State University, Columbus, OH 43210, USA; Department of Medicine, Baylor College of Medicine, Houston, TX 06520, USA; Machine Learning Department, NEC Labs America, Princeton, NJ 08540, USA

## Abstract

**Motivation:**

MHC Class I protein plays an important role in immunotherapy by presenting immunogenic peptides to anti-tumor immune cells. The repertoires of peptides for various MHC Class I proteins are distinct, which can be reflected by their diverse binding motifs. To characterize binding motifs for MHC Class I proteins, *in vitro* experiments have been conducted to screen peptides with high binding affinities to hundreds of given MHC Class I proteins. However, considering tens of thousands of known MHC Class I proteins, conducting *in vitro* experiments for extensive MHC proteins is infeasible, and thus a more efficient and scalable way to characterize binding motifs is needed.

**Results:**

We presented a de novo generation framework, coined PepPPO, to characterize binding motif for any given MHC Class I proteins via generating repertoires of peptides presented by them. PepPPO leverages a reinforcement learning agent with a mutation policy to mutate random input peptides into positive presented ones. Using PepPPO, we characterized binding motifs for around 10 000 known human MHC Class I proteins with and without experimental data. These computed motifs demonstrated high similarities with those derived from experimental data. In addition, we found that the motifs could be used for the rapid screening of neoantigens at a much lower time cost than previous deep-learning methods.

**Availability and implementation:**

The software can be found in https://github.com/minrq/pMHC.

**Supplementary information:**

Supplementary data are available at *Bioinformatics* online.

## 1 Introduction

Immune responses can be triggered when T cells recognize immunogenic peptides presented by Major Histocompatibility Complex (MHC) Class I proteins on the surface of infected or malignant cells (Hennecke and Wiley, 2001). These immunogenic peptides are typically degraded from intracellular antigens, and bind to MHC Class I proteins; the resulting peptide-MHC complexes are then moved to the cell surface to interact with the CD8+ T cell receptors (Craiu *et al.*, 1997). For this reason, peptides are promising therapeutic targets and have been used as personalized vaccines in the prevention of human diseases (Duperret *et al.*, 2019; Roozbehani *et al.*, 2018).

The binding process between peptides and MHC I proteins is highly specific, largely depending on the compatibility between motifs of peptide sequence and the structure of MHC I binding grooves. Characterizing binding motifs for MHC Class I proteins requires statistically summarizing conservative residues from a large number of peptides with high binding affinities to given MHC Class I proteins. However, due to the high cost and workload, experimental results only cover a few hundred MHC I proteins, leaving thousands of known MHC I proteins without data (Rasmussen *et al.*, 2014; Marco *et al.*, 2017). To address this issue, an alternative approach is to search for high-binding peptides using *in silico* computational methods for peptide binding predictions [e.g. NetMHCPan-4.1 (Reynisson *et al.*, 2020) and MHCflurry2.0 (O’Donnell *et al.*, 2020)]. Yet, searching for high-binding peptides one by one using these prediction methods for a given MHC I protein is still inefficient due to the sparsity of binding peptides over a large peptide space. In order to tackle this challenge, we propose to directly generate a repertoire of binding peptides for any given MHC protein and identify binding motifs from the generated data.

We formulated the binding peptides generation as a reinforcement learning (RL) problem and proposed a framework, named PepPPO, to generate qualified peptides for binding motif characterization. Leveraging the RL agent and the rewards from peptide-MHC binding predictor (i.e. MHCflurry2.0), our PepPPO learns a mutation policy to optimize random initial peptides through mutating amino acids step by step until the mutated peptides can be predicted to be positive and thus very likely to be presented by a given MHC protein. These mutated peptides can be directly used for motif characterization amid insufficient experimental data. We found that the motifs characterized from generated peptides by PepPPO are highly correlated with experimentally derived motifs; these motifs are also highly robust to changes in random initial peptides, indicating that PepPPO can consistently mutate different random initial peptides into binding peptides following the identical motifs. In addition, the motifs computed by PepPPO are demonstrated to be effective in the rapid screening of neoantigens for human MHC Class I proteins with and without experimental data. Furthermore, PepPPO significantly outperformed multiple baselines in generating qualified peptides.

## 2 Materials and methods

### 2.1 Problem definition and formulation

In this article, we represent a peptide as a sequence of amino acids $<o_{1},o_{2},\ldots ,o_{i},\ldots ,o_{l}>$, where *o* is one of 20 types of natural amino acids and l is the length of the sequence ranging from 8 to 15. Given an MHC protein *m* that is another sequence of amino acids, PepPPO aims at generating a binding peptide *p* of length *l* that will be presented by *m*.

**Fig. 1.**
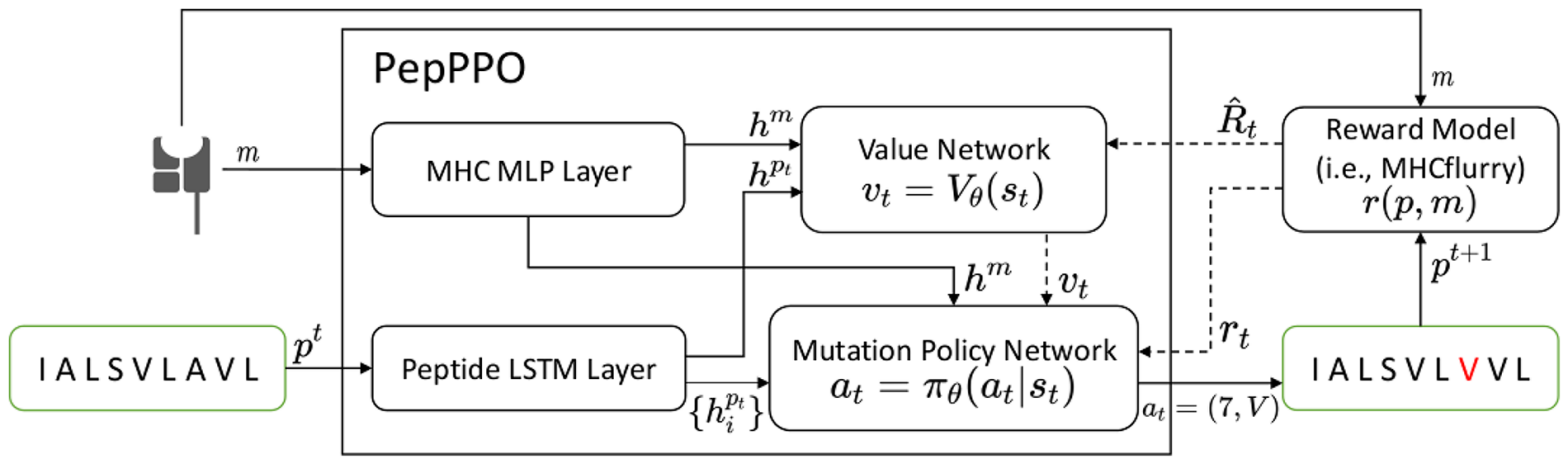
Model Architecture of PepPPO. Dashed arrows represent that the values are transmitted to optimize the value network/mutation policy network

To this end, PepPPO leverages a reinforcement learning (RL) agent to explore (interact with) the peptide mutation environment for high-presentation peptide generation. In a nutshell, given a peptide and an MHC protein pair (*p*, *m*), the RL agent explores and exploits the peptide mutation environment by repeatedly mutating the current peptide and observing its presentation score. Through such trial-and-error processes, the agent learns to form a mutation policy π(.) to iteratively mutate the amino acids of any given peptide *p* to have a desired presentation score. This learning paradigm is illustrated in Figure 1, and there are two main components to fulfill the learning: constructing the peptide mutation environment and learning the mutation policy network, which will be discussed next.

Here, the peptide mutation environment enables the RL agent to perform and experience trial-and-error peptide mutations to gradually refine its mutation policy (through tuning the parameters of the mutation policy networks in our case). During learning, the RL agent keeps mutating peptides and receiving their presentation scores (i.e. reward signal) given by the environment. These rewards thus help reinforce the agent’s mutation behaviors: mutation behaviors resulting in high peptide presentation scores (high rewards) are encouraged while others leading to low scores are discouraged.

### 2.2 Peptide mutation environment

The mutation environment consists of three components: the state space, the action space and the reward function. The state includes the current mutated peptide and the MHC protein. The action and the reward represent the mutation action that may be taken by the RL agent and the resulting new presentation score of the mutated peptide, respectively.

#### 2.2.1 State space

We define the state of the environment *s_t_* at time step *t* as a pair consisting of a peptide and an MHC Class I protein (*p*, *m*). We represent an MHC protein as a pseudo sequence with 34 amino acids, each of which is in potential contact with the bound peptide within a distance of 4.0 Å, following the previous works for peptide-MHC binding prediction (Nielsen *et al.*, 2007; Jurtz *et al.*, 2017). With a peptide of length *l* and an MHC protein, we represent the state *s_t_* as a tuple $s^{t}=(E^{p},E^{m})$, in which *E^p^* and *E^m^* are the encoding matrices of the peptide and the MHC molecule, respectively. For training, we initialize the state *s*_0_ by an MHC Class I protein and a peptide sequence. We define the terminal state *s_T_*, which will stop mutating a peptide, as the state either with the maximum time step *T* or with the presentation score greater than threshold *σ*.

#### 2.2.2 Action space

We define a multi-discrete action space to optimize the peptide by replacing one amino acid with another one. At time step *t*, given a peptide $p^{t}=<o_{0},o_{2},\ldots ,o_{l}>$, the action for the RL agent is to first determine the position of the amino acid to be replaced, and then predict the type of new amino acid at that position.

#### 2.2.3 Reward design

We use the final reward to guide the optimization of the RL agent. That is, only the terminal states can receive rewards from the peptide mutation environment. We define the final reward as the presentation score *r*(*p*, *m*) between the peptide *p_T_* and the MHC protein *m* in the terminal state *s_T_*. To this end, we leverage the presentation score predicted by the MHCflurry2.0 (O’Donnell *et al.*, 2020) for learning. MHCflurry2.0 is the best existing method able to accurately estimate the presentation scores of peptides with MHC proteins. This score is a composite score of the antigen processing (AP) prediction and the binding affinity (BA) prediction. The former predicts the probability for a peptide to be delivered by the transporter associated with antigen processing (TAP) protein complex into the endoplasmic reticulum (ER), where the peptide can bind to MHC proteins. The latter predicts the binding strength between the peptide and MHC protein. Higher presentation scores require higher AP and BA scores, and indicate higher probabilities for peptides to be presented on the cell surface by the given MHC proteins. Detailed discussions about the choice of MHCflurry2.0 is available in Supplementary Section S3.

### 2.3 Mutation policy network

To learn the mutation policy, the RL agent in the PepPPO takes as input the given peptide and the MHC protein. The agent then learns to mutate the amino acids in the peptide sequence, one amino acid at each step, aiming at maximizing the presentation score of the resulting peptide. In PepPPO, both the peptide and the MHC protein are first encoded into a distributed embedding space. Then, a mapping between the embedding space and the mutation policy is learned by a gradient descent optimization method, as discussed next.

#### 2.3.1 Encoding of amino acids

We use a mixture of multiple encoding methods to represent the amino acids within the peptide sequences and the MHC molecules.

We represent each amino acid by concatenating the encoding vectors $e^{B},\;e^{O}$ and $e^{D}$ from the BLOSUM matrix (Henikoff and Henikoff, 1992), the one-hot matrix and the learnable embedding matrix, respectively, that is, $e=e^{B}⊕e^{O}⊕e^{D}$ and $e\in R^{d}$. This method has been demonstrated in Chen *et al.* (2021) to achieve the best prediction performance on peptide-MHC binding prediction among all the combinations of these encoding methods. The encoding matrices *E^p^* and *E^m^* of the peptide *p* and the MHC molecule *m* are then represented as $E^{p}=\{e_{1},\ldots ,e_{l}\}\in R^{l\times d}$ and $E^{m}=\{e_{1},\ldots ,e_{34}\}\in R^{34\times d}$, respectively.

#### 2.3.2 Embedding of states

In order to predict the mutation of amino acids in peptide sequences, we first embed each amino acid *o_i_* within the peptide sequences $<o_{0},o_{2},\ldots ,o_{l}>$ into an continuous latent vector $h_{i}$ using one-layer bidirectional LSTM (Graves and Schmidhuber, 2005) as below:

(1) $$\begin{array}{c} {\vec{h}}_{i},{\vec{c}}_{i}=\text{LSTM}(e_{i},{\vec{h}}_{i-1},{\vec{c}}_{i-1};{\vec{W}}^{p})\\ {\overset{\leftarrow }{h}}_{i},{\overset{\leftarrow }{c}}_{i}=\text{LSTM}(e_{i},{\overset{\leftarrow }{h}}_{i+1},{\overset{\leftarrow }{c}}_{i+1};{\overset{\leftarrow }{W}}^{p})\\ h_{i}={\vec{h}}_{i}\;⊕\;{\overset{\leftarrow }{h}}_{i} \end{array}$$

where ${\vec{h}}_{i}/{\overset{\leftarrow }{h}}_{i}$ is the hidden state vector of *i*th amino acid; $\vec{c}/\overset{\leftarrow }{c}$ are the memory cell states of *i*th amino acid; ${\vec{h}}_{0},\;{\overset{\leftarrow }{h}}_{l},\;{\vec{c}}_{0}$ and ${\overset{\leftarrow }{c}}_{l}$ are initialized with random noise vectors; ${\vec{W}}^{p}$ and ${\overset{\leftarrow }{W}}^{p}$ are the learnable parameters of LSTM of forward and backward direction, respectively. With the embeddings of all the amino acids, we define the embedding of the peptide sequence as the concatenation of hidden vectors at two ends, that is, $h^{p}=\vec{h_{l}}\;⊕\;\overset{\leftarrow }{h_{0}}$.

To embed an MHC protein into a continuous latent vector, we first flatten the encoding matrix $E^{m}$ into a vector **m**. Then, we learn the continuous latent embedding $h^{m}$ with,

(2) $$h^{m}=W_{1}^{m}\text{ReLU}(W_{2}^{m}m)$$

where $W_{i}^{m}$(*i* = 1,2) are the learnable parameter matrices.

#### 2.3.3 Action prediction

At time step *t*, we optimize the peptide sequence *p_t_* by predicting the mutation of one amino acid with the latent embeddings $h^{p_{t}}$ and $h^{m}$. Specifically, we first select the amino acid *o_i_* in *p_t_* as the one to be replaced. We then predict which amino acid should be used to replace *o_i_*. For each amino acid *o_i_* in the peptide sequence, we predict the score of replacement as below:

(3) $$f^{c}(o_{i})={\left(w^{c}\right)}^{T}(\text{ReLU}(W_{1}^{c}h_{i}+W_{2}^{c}h^{m}))$$

where $h_{i}$ is the hidden latent vector of amino acid *o_i_* from LSTM; $w^{c}$ and $W_{i}^{c}$ (*i* = 1,2) are the learnable scalar, vector and matrices, respectively. We measure ‘how likely’ the amino acid *o_i_* can be replaced with another one by looking at its context in $h_{i}$ (i.e. *o_i_* and the peptide sequence *p*) and the MHC protein $h^{m}$. The amino acid to be replaced is determined by sampling from the distribution with normalized scores. We then predict the type of the amino acid used to replace *o_i_* as below:

(4) $$f^{d}(o_{i})=\text{softmax}(W_{1}^{d}\times \text{ReLU}(W_{2}^{d}h_{i}+W_{3}^{d}h^{m})),$$

where $W_{i}^{d}$ (*i* = 1,2,3) are the learnable matrices; softmax(.) converts a vector into probabilities over 20 amino acid types. The amino acid type is then determined by sampling from the distribution of probabilities of amino acid types excluding the original type of *o_i_*

### 2.4 Learning

#### 2.4.1 Optimization

We adopt Proximal Policy Optimization (PPO) (Schulman *et al.*, 2017), a widely used policy gradient method, to optimize the policy networks as discussed in Section 2.3. Specifically, in each iteration, we collect *N* time steps data by applying the policy networks to modify peptides. We optimize the policy networks using the collected data for *K* epochs. The objective function of PPO is defined as below:

(5) $$\max L^{\mathrm{CLIP}}(\theta )={\hat{E}}_{t}[\min(\phi_{t}(\theta ){\hat{A}}_{t},\text{clip}(\phi_{t}(\theta ),1-\epsilon ,1+\epsilon ){\hat{A}}_{t})]$$

where $\phi_{t}(\theta )=\frac{\pi_{\theta }(a_{t}|s_{t})}{\pi_{\theta_{\mathrm{old}}}(a_{t}|s_{t})}$ is the probability ratio between the action under current policy $\pi_{\theta }$ and the action under previous policy $\pi_{\theta_{\mathrm{old}}}$. $\phi_{t}(\theta )$ is clipped to avoid moving $\phi_{t}$ outside of the interval [1−ϵ,1+ϵ]. ${\hat{A}}_{t}$ is an estimator of advantage function at time step *t* computed with the generalized advantage estimator (Schulman *et al.*, 2016), measuring how much better the selected actions are than others on average:

(6) $${\hat{A}}_{t}=\delta_{t}+(\gamma \lambda )\delta_{t+1}+\cdots +{\left(\gamma \lambda \right)}^{T-t+1}\delta_{T-1}$$

(7) $$\text{where }\delta_{t}=r_{t}+\gamma V_{\theta }(s_{t+1})-V_{\theta }(s_{t})$$

where *γ* is a hyper-parameter (i.e. discount factor) determining the importance of future rewards; *λ* is a hyper-parameter used to control the trade-off between bias and variance of advantages; $V_{\theta }(s_{t})$ is an MLP-based value function that estimates the future return of current state *s_t_*; *δ_t_* is the temporal difference error; *r_t_* is the reward. In particular, $V_{\theta }(s_{t})$ takes the latent embeddings $h^{m}$ and $h^{p}$ for the MHC and peptide in *s_t_*. The objective function of $V_{\theta }(.)$ is defined as below:

(8) $$\min L^{V}(\theta )={\hat{E}}_{t}[{\left(V_{\theta }(s_{t})-{\hat{R}}_{t}\right)}^{2}]$$

where ${\hat{R}}_{t}=\sum_{i=t+1}^{T}\gamma^{i}r_{i}$ is the rewards-to-go. Because we only use the final rewards, that is $r_{i}=0\;\text{if}\;i\neq T$, we calculate ${\hat{R}}_{t}$ with ${\hat{R}}_{t}=\gamma^{T-t}r_{T}$. We also add the entropy regularization H(θ) to encourage the policy to produce diverse actions. The learning algorithm of PepPPO is presented in Supplementary Algorithm S2 in Supplementary Information.

#### 2.4.2 Informative training with prior knowledge

In order to stabilize the training and improve the performance, we derive an expert policy *π_ept_* from the existing data. Specifically, for each MHC molecule *m* with enough data, we calculate the amino acid distributions $<p_{1}(o|m),p_{2}(o|m),\ldots ,p_{l}(o|m)>$ of peptides with length *l*. Given a peptide $p=<o_{1},o_{2},\ldots ,o_{l}>$, we select the position *i* as follows,

(9) $$\pi_{\mathrm{ept}}^{c}(p,m)=\arg\max_{i}(p_{i}(o={\hat{o}}_{i}|m)-p_{i}(o=o_{i}|m)),$$

where ${\hat{o}}_{i}$ is the most popular amino acid on position *i*, that is, $p_{i}(o={\hat{o}}_{i}|m)=\max_{o}(p_{i}(o|m))$. After determining the position, we sample the amino acid from the distribution $o_{i}^{\prime }∼p_{i}(o|m)$. For an MHC protein without experimental data, we calculate its distances with all the MHCs with data using the BLOSUM62 matrix, and sample actions from the amino acid distributions of the most similar MHC.

Similar to (Silver *et al.*, 2016), we utilize the expert policy to pre-train the policy network. The objective of pre-training is to minimize the following cross entropy loss,

(10) $$\begin{array}{c} \min L^{\mathrm{PRE}}(\theta )=E_{s∼S}(E_{i∼\pi_{\mathrm{ept}}^{c}}(\text{log}(\pi_{\theta }^{c}(i|s)))\\ +E_{o∼\pi_{\mathrm{ept}}^{d}}(\text{log}(\pi_{\theta }^{d}(o|s)))) \end{array}.$$

where *S* denotes the state space. In addition to pre-training the policy network, at the beginning of training, we also sample actions with the expert policy, and use the trajectories with expert actions to update the policy network.

#### 2.4.3 Diversity-promoting experience buffer

To increase the diversity of generated peptides, it is important to find a non-deterministic policy that could produce diverse actions. Such a policy can increase the exploration over a large state space and thus find diverse good actions.

As mentioned earlier, we have included the entropy regularization into our objective function to ensure sufficient exploration. However, this strategy cannot explicitly encourage the policy to produce diverse actions that could lead to high rewards. To explicitly enforce the policy to learn diverse actions, we design a diversity-promoting experience buffer to store the trajectories that could result in qualified peptides. In detail, at each iteration, we add the visited state-action pairs of mutation trajectories of qualified peptides into this buffer. We always keep the state-action pairs with infrequent actions and remove those with frequent actions to ensure that the buffer is not dominated by the frequent actions. We then randomly sample a batch of state-action pairs with infrequent actions from the buffer. To encourage the policy network to reproduce these infrequent actions that could induce high rewards, we define the cross-entropy loss *L^B^* as below:

(11) $$\begin{array}{c} L^{B}=E_{(s,i,o\prime )∼B}[-\sum_{j=1}I(j=i)\;\text{log}(\pi_{\theta }^{c}(i|s))\\ -\sum_{o_{i}}I(o_{i}=o\prime )\;\text{log}(\pi_{\theta }^{d}(o_{i}|s))] \end{array}.$$

where *B* represents the diversity-promoting experience buffer; *I* represents the indicator function. We then include the above object function into the final objective function as below:

(12) $$\underset{\theta }{\min}L(\theta )=-L^{\text{CLIP}}(\theta )+\alpha_{1}L^{V}(\theta )+\alpha_{2}L^{B}(\theta )-\alpha_{3}H(\theta ),$$

where $\alpha_{\ell }(\ell =1,2,3)$ are the pre-defined coefficients.

### 2.5 Dataset

The experimental dataset of MHC binding affinities (O’Donnell, 2020) was used to derive the amino acid distributions of qualified peptides to get the expert policy for PepPPO. This dataset contains 149 human MHC Class I proteins (alleles) and 309 963 peptides. We also collected 3 688 unique pseudo sequences for 10 402 MHC proteins from a previous publication (Jurtz *et al.*, 2017), and trained PepPPO to optimize the peptides toward them. Note that different MHC proteins could be represented with the same pseudo sequences.

### 2.6 Comparison with baseline methods

Five baselines, shown below, are developed for comparison (The details of how these methods are implemented can be found in Supplementary Section S1):

- MCTS: Monte Carlo Tree Search (Coulom, 2007)
- BO-VAE: Bayesian Optimization with the Variational Autoencoder (VAE) (Kingma and Welling, 2014)
- BP-VAE: Back Propagation with a VAE
- sPWM: Sampling from Position Weight Matrix.
- Random: Randomly generating peptide sequences of length from 8 to 15.

As mentioned in Section 2.2.1, we represent each MHC Class I protein with its pseudo sequence in the peptide-contacting positions. It is noted that for 95.66% of the MHC pseudo sequences, we do not have any experimental data; therefore, it is impossible to apply traditional conditional generative adversarial networks (Mirza and Osindero, 2014) or conditional VAE (Sohn *et al.*, 2015) to this problem.

We selected 30 common (with experimental data) and 30 rare MHC proteins (without experimental data) and used each method to generate 1000 peptides for each protein. Within each group of 30 common or rare proteins (Supplementary Table S1), 10 MHC proteins from each of the three groups: HLA-A, HLA-B and HLA-C are included. We used MHCflurry2.0 to calculate the presentation score for each peptide and compare PepPPO with the five baseline methods in terms of the percentage of qualified peptides (presentation scores above 0.75), average and maximum presentation scores. The hyperparameter setup of PepPPO can be found in Supplementary Section S2 and Supplementary Table S2.

### 2.7 Motif characterization

Using PepPPO, we generated 1000 peptides for each MHC protein and calculated position weight matrices (PWMs) to represent the binding motifs. To exclude low presentation peptides, we used MHCflurry2.0 to calculate presentation scores of generated peptides for given MHC alleles and filter out those with scores below 0.75. In total, PepPPO supports 10 402 MHC proteins. We visualized 9-mer binding motifs using t-SNE plots based on distances between them. The distance is measured by averaging the Hellinger distances of columns from two PWMs.

In addition to the motifs, we also visualized the distances between pseudo sequences of these MHC alleles using t-SNE plots. More specifically, we calculated the similarities between them based on BLOSUM62 matrix, which are later normalized to range 0–1. The distances are then calculated by using one minus the similarities.

### 2.8 Motif robustness

To evaluate the robustness of the motifs computed by PepPPO, we used PepPPO to generate 1000 peptides with random initialization for the aforementioned 30 MHC proteins 5 times. To compare results from different runs, we calculated distances between binding motifs as previously described. Similarity scores are then calculated by subtracting the distances from one. We selected a common allele (HLA-B40:02) and a rare allele (HLA-B54:38) to visualize the computed motifs from the 5 runs using sequence logo plots.

### 2.9 Correlation between computed and real motifs

We characterized real motifs using experimental data (O’Donnell, 2020), containing 149 human MHC proteins and 309 963 peptides. For computed motifs, we used PepPPO to generate 1000 peptides for each of the 149 MHC proteins. Generated peptides with presentation scores below 0.75 are excluded due to low binding affinity. The similarity scores between real and computed motifs are calculated as described in section 2.8.

### 2.10 Neoantigen characterization

Mutation annotation files of 69 Rectum adenocarcinoma (READ) patients from The Cancer Genome Atlas (TCGA) are downloaded from FireBrowse (http://firebrowse.org). The MHC I allele haplotype for each patient is retrieved from a previous publication (Charoentong *et al.*, 2017). In total, 99 MHC alleles are included. We mapped the DNA changes to protein sequence mutation, and slided windows with 9 amino acids of length along the mutation sites to select all possible peptides. Using MHCflurry2.0, we calculated a presentation score of each peptide to a specific MHC allele for each patient. Those with scores above 0.75 were selected as candidate neoantigens. Besides MHCflurry2.0, we also applied NetMHCPan-4.1 as an additional method to characterize neoantigens for further evaluation of computed motifs. We considered strong binders predicted by NetMHCPan 4.1 as neoantigens with the default threshold (%Rank < 0.5).

### 2.11 Motif matching score

To examine the ability of computed motifs for screening neoantigens, we calculated motif matching scores between pairs of motifs and peptides. Specifically, we calculated a position weight matrix (PWM) for each MHC allele using generated peptides from PepPPO. Each PWM is a matrix with the shape of 20 × length, in which each element corresponds to the probability of a specific amino acid on a specific position in the binding peptides. To calculate the matching score between a given peptide and the MHC I allele, we extracted from PWM the probabilities of amino acids in the given peptide appearing in the corresponding positions, and summed over the probabilities as the matching score. The matching score is further min-max normalzied into [0,1] range. The two-sided Wilcoxon test is used to compare the motif matching scores between neoantigens and non-neoantigens. Area under receiver operating characteristic curve (AUC) is used to evaluate the performance of motif matching score in discriminating neoantigens from non-neoantigens.

## 3 Results

### 3.1 PepPPO shows high performance in generating qualified peptides

To demonstrate the effectiveness of PepPPO, we developed five baselines for comparison (Table 1). Table 1 presents the overall performance among all the methods on generating qualified peptides with respect to 30 MHC proteins (Supplementary Table S1) with (i.e. common MHCs) and without (i.e. rare MHCs) experimental data. These 30 common or rare MHC proteins include 10 MHC proteins from each of the three groups: HLA-A, HLA-B and HLA-C. We utilized each method to generate 1000 peptides for each MHC protein.

**Table 1.** Overall performance comparison

MHC	Method	Percentage (%)	Avg score	Max score
Common	BO-VAE	3.37 ± 2.53	0.13 ± 0.23	0.97 ± 0.03
	BP-VAE	1.85 ± 1.26	0.06 ± 0.16	0.95 ± 0.07
	MCTS	13.90 ± 9.34	0.16 ± 0.29	0.96 ± 0.02
	sPWM	30.60 ± 12.78	0.40 ± 0.39	0.99 ± 0.01
	Random	0.43 ± 0.36	0.02 ± 0.09	0.89 ± 0.10
	PepPPO (w/o buffer)	**91.48 ± 10.68**	0.83 ± 0.17	0.99 ± 0.01
	PepPPO (w buffer)	91.42 ± 10.89	0.83 ± 0.17	0.99 ± 0.00
Rare	BO-VAE	2.59 ± 3.41	0.11 ± 0.21	0.93 ± 0.06
	BP-VAE	1.34 ± 1.24	0.05 ± 0.14	0.92 ± 0.09
	MCTS	8.11 ± 7.11	0.11 ± 0.23	0.94 ± 0.03
	sPWM	18.80 ± 8.99	0.28 ± 0.35	0.98 ± 0.01
	Random	0.26 ± 0.34	0.02 ± 0.07	0.83 ± 0.15
	PepPPO (w/o buffer)	85.33 ± 14.09	0.78 ± 0.21	0.98 ± 0.01
	PepPPO (w buffer)	**87.15 ± 9.71**	0.79 ± 0.20	0.98 ± 0.01

*Note*: We present the mean and standard deviation over the 30 MHC proteins. Columns represent: ‘Percentage (%)’ denotes the percentage of qualified peptides among all the generated peptides; ‘Avg Score’ denotes the average presentation scores of generated peptides; ‘Max Score’ denotes the maximum presentation scores among the generated peptides. Best percentage (%) values are in bold.

Table 1 shows that the PepPPO, both with and without the diversity-promoting buffer, achieves the best performance among all the methods in terms of percentage of qualified peptides (i.e. with presentation scores greater than 0.75), average presentation scores and maximum presentation scores among the generated peptides.

For example, for common MHC proteins, PepPPO with the buffer outperforms the second best model sPWM by 60.82% (i.e. 91.42% for PepPPO versus 30.60% for sPWM) on average. The suboptimal performance of sPWM on these MHC proteins demonstrates that the amino acid distributions derived from the dataset can describe the distribution of qualified peptides of MHC proteins. Also, the superior performance of PepPPO indicates that PepPPO may learn the additional patterns of qualified peptides that are not captured by sPWM.

When considering rare MHC proteins, PepPPO with the buffer significantly outperforms the best baseline sPWM by 68.35% (i.e. 87.15% for PepPPO versus 18.80% for sPWM). Our further analysis suggests that when the MHC proteins do not have enough qualified peptides, the amino acid distributions from the most similar MHC proteins can fail to accurately describe the distribution of qualified peptides, leading to sPWM’s distinctly worse performance in generating qualified peptides for rare MHCs when compared to common MHCs. On the contrary, PepPPO learns a common policy network to generate peptides presented by any MHC protein and thus only achieves only slightly worse performance on rare MHCs than that on common MHCs.

### 3.2 Characteristics of binding motifs generated

The MHC protein’s specificity on binding peptides can be reflected by peptide binding motifs, which contain residues at specific positions that can interact with binding grooves of given MHC proteins. However, current experimental data only cover a limited number of MHC alleles. Given tens of thousands of known human MHC I alleles, most binding motifs can’t be directly characterized. As previously described, PepPPO can efficiently generate peptides presented by given MHC alleles, hence enabling us to characterize binding motifs for a large number of rare alleles without experimental data.

**Fig. 2.**
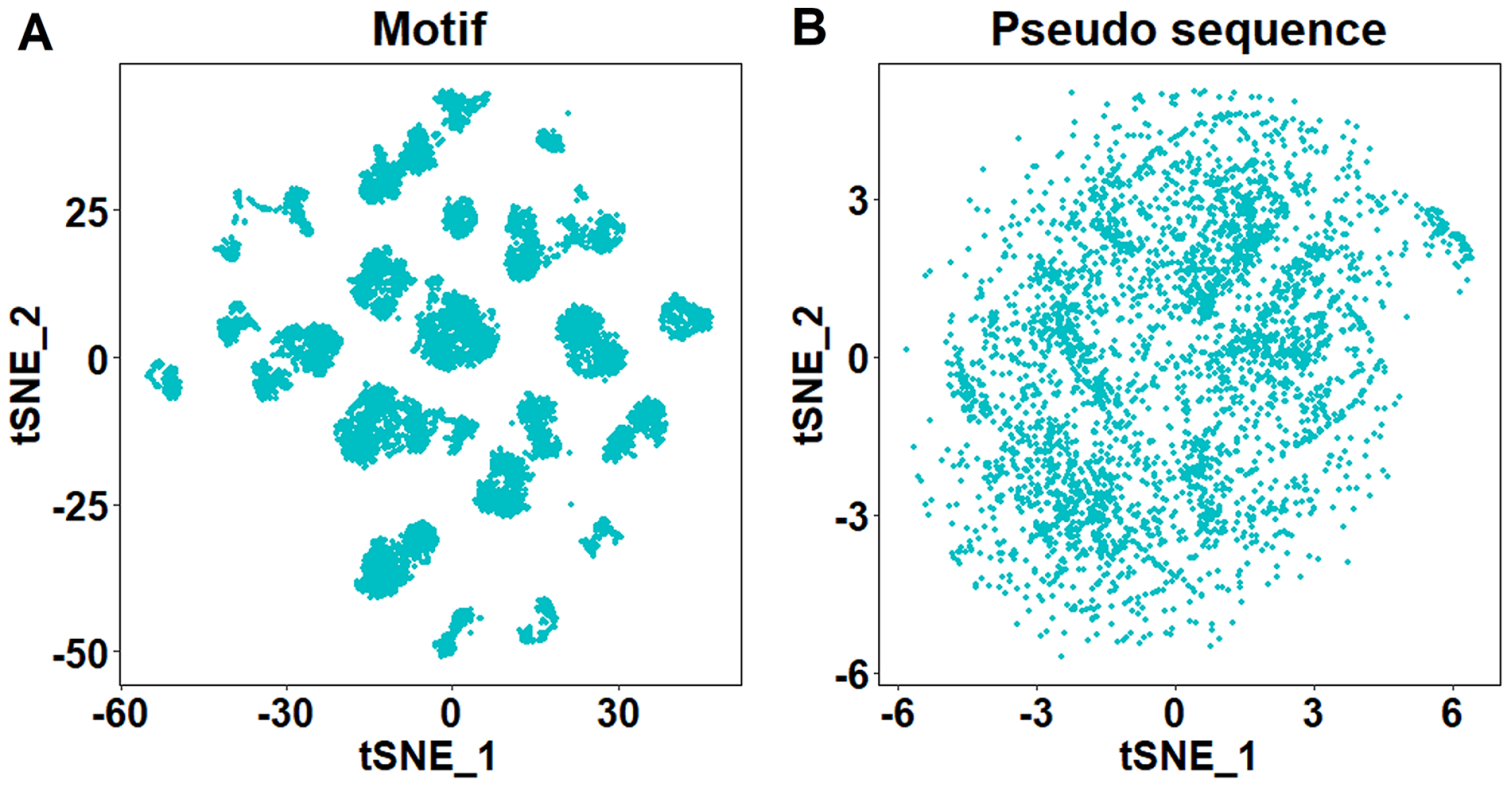
t-SNE plots of the 10402 9-mer motifs (**A**) and 3688 corresponding pseudo sequences (**B**)

We characterized motifs for 10 402 MHC alleles using PepPPO. Specifically, we used PepPPO to generate 1000 peptides per MHC allele and calculate the PWM to represent the binding motif. The 10 402 binding motifs for 9-mer peptides were visualized in Figure 2A as t-SNE plots. When compared to the t-SNE plots of pseudo sequences from these MHC alleles, the binding motifs tend to form more distinct clusters. This result indicates that through training with experimental data, the model can learn additional information about how peptides interact with MHC alleles.

**Fig. 3.**
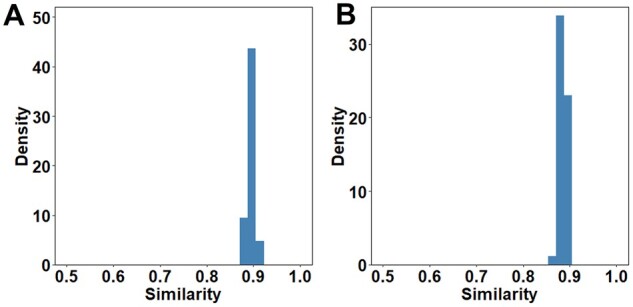
The distributions of the similarities between 9-mer motifs from 5 runs of PepPPO. The pairwise similarities were calculated among the five runs of the same allele. In total, 30 common (**A**) and 30 rare alleles (**B**) were examined (Supplementary Table S1)

We next evaluated the robustness of these computed motifs. To this end, we took the aforementioned 60 MHC alleles (30 common and 30 rare alleles) and generated 1000 peptides with random initial states for each allele 5 times using PepPPO. As shown in Figure 3, the 9-mer motifs derived from 5 runs of PepPPO on the same allele are highly consistent, with an average similarity of 0.89 for both common and rare alleles. Similar results were observed for 8 to 15-mer motifs (Supplementary Fig. S1A and B). Taking two alleles (HLA-B40:02 and HLA-B54:38) as an example, we visualized their motifs derived from the five runs in Supplementary Figure S2A and B. As shown, the sequence logos are highly similar for the same alleles. These results suggest that computed motifs are robust and not affected by the sequences of initial peptides.

**Fig. 4.**
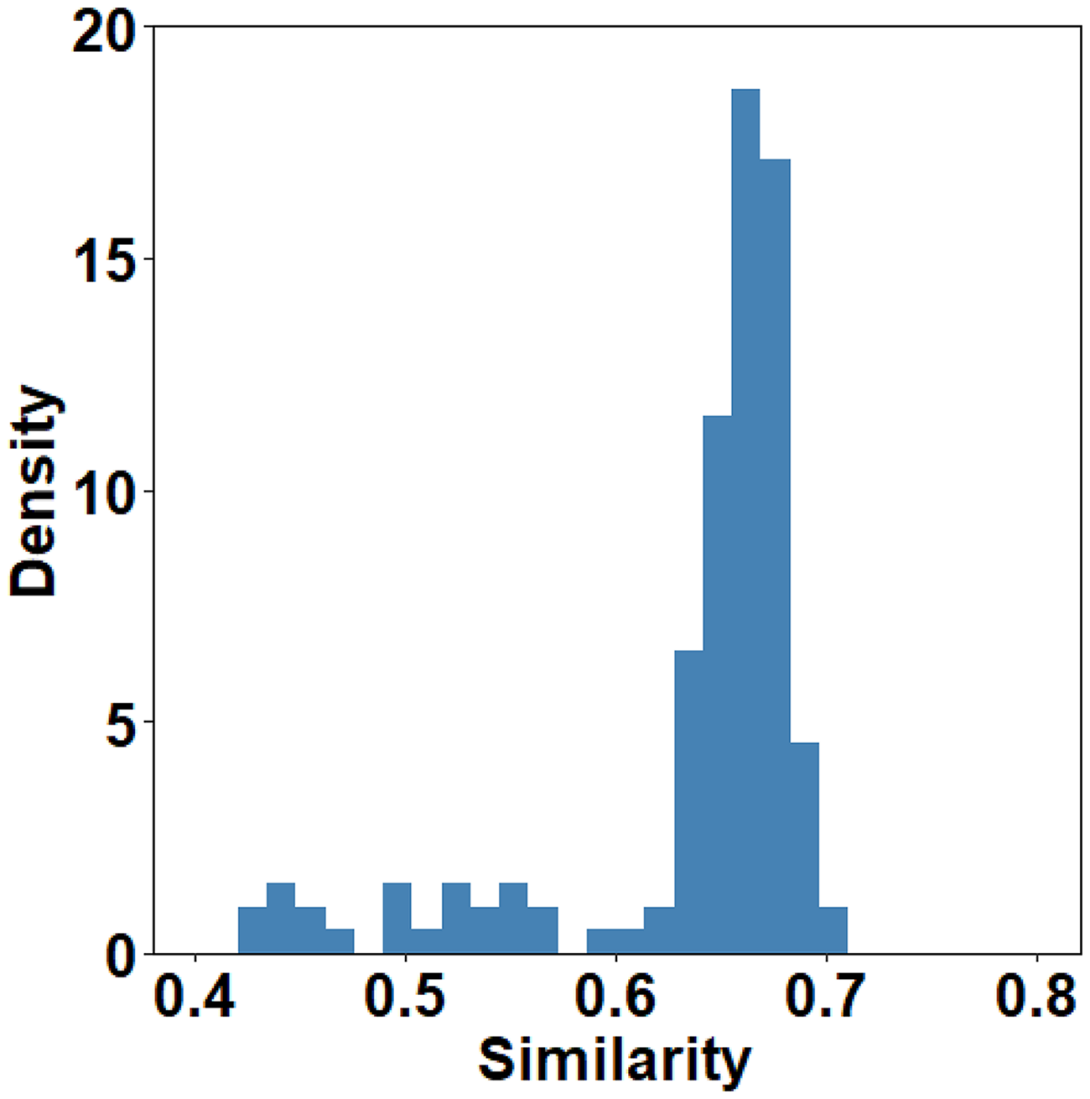
The distribution of the similarities between computed and real motifs for 9-mer peptides

**Fig. 5.**
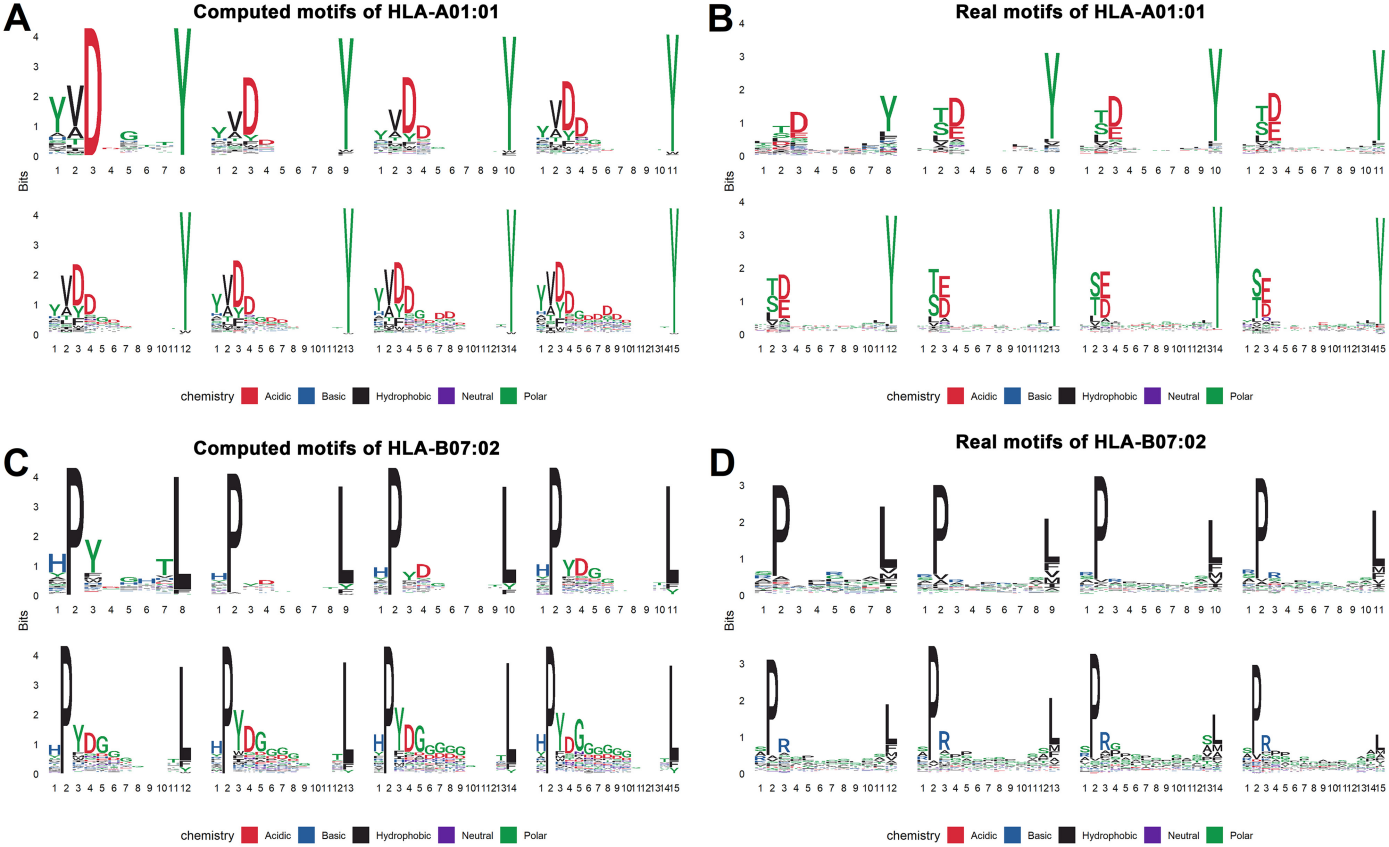
The computed motifs are highly correlated with real motifs derived from experimental data. We took HLA-A01:01 (**A**, **B**) and HLA-B07:02 (**C**, **D**) as examples to visualize the correlation between the computed and real motifs

In addition, we examined if the computed motifs are correlated with the real motifs derived from experimental data. We downloaded experimental data containing 149 human MHC I alleles with binding affinity values of corresponding peptides from a previous publication (O’Donnell et al., 2020). The distribution of similarities between computed and real motifs for 9-mer peptides are shown in Figure 4. As shown, 83% of the alleles have similarities over 0.6, with an average similarity of 0.63. Compared to similarities between different runs of PepPPO, similarities between computed and real motifs are inferior, potentially due to more variations allowed at non-anchor residues in the real world. We observed similar results in motifs for 8 to 15-mer peptides, with average similarities of 0.49, 0.66, 0.68, 0.68, 0.65, 0.68 and 0.69, respectively (Supplementary Fig. S3). Note that similarities for motifs of 8-mer peptides are relatively lower than those of longer peptides, as the computed motifs are more conserved than the real motifs. We found that less percentage of 8-mer binding peptides will be predicted to be positive, as 8-mer binding peptides in experimental data have lower presentation scores on average than longer peptides (i.e. 0.4537 for 8-mer versus 0.7926, 0.6220 and 0.5119 for 9-mer, 10-mer and 11-mer, respectively). Therefore, the generated positive 8-mer peptides could be limited to less binding patterns with large presentation scores, leading to more conserved computed motifs for 8-mers. Taking HLA-A01:01 and HLA-B07:02 MHC proteins as examples, we also visualized their real and computed motifs in Figure 5. This figure shows that the computed motifs from PepPPO are very similar with the real motifs; these motifs for peptides of different lengths binding to the same MHC protein share the similar conservation patterns on specific positions.

Taken together, PepPPO computed motifs are not only robust but are also correlated with real world data, making PepPPO an appealing solution to clinical applications with inadequate data since characterizing the binding motif of a given peptide requires costly experimental work. Next, we will demonstrate such potential.

### 3.3 Screening neoantigens with generated motifs

To explore the clinical application of PepPPO, we examined whether computed motifs by PepPPO can be used to rapidly screen neoantigens while achieving consistent results with peptide-MHC binding predictors (e.g. MHCflurry2.0). We utilized MHCflurry2.0 to identify neoantigens from 9-mer mutated peptides for each patient in TCGA READ cohorts. Specifically, we considered those with presentation scores above 0.75 as neoantigens. Next, we calculated a PWM to represent the binding motif for each allele based on 1000 peptides generated from PepPPO. Therefore, given a pair of allele and peptide, we can calculate a motif matching score by adding up the amino acid values at specific positions within the PWM. A high matching score indicates that a given peptide tends to be presented by a given allele, thereby being a potential neoantigen. Using these matching scores without deep learning-based frameworks should be much faster in screening neoantigens, so we then examined the performance of these matching scores in distinguishing neoantigens from non-neoantigens identified by MHCflurry2.0.

**Fig. 6.**
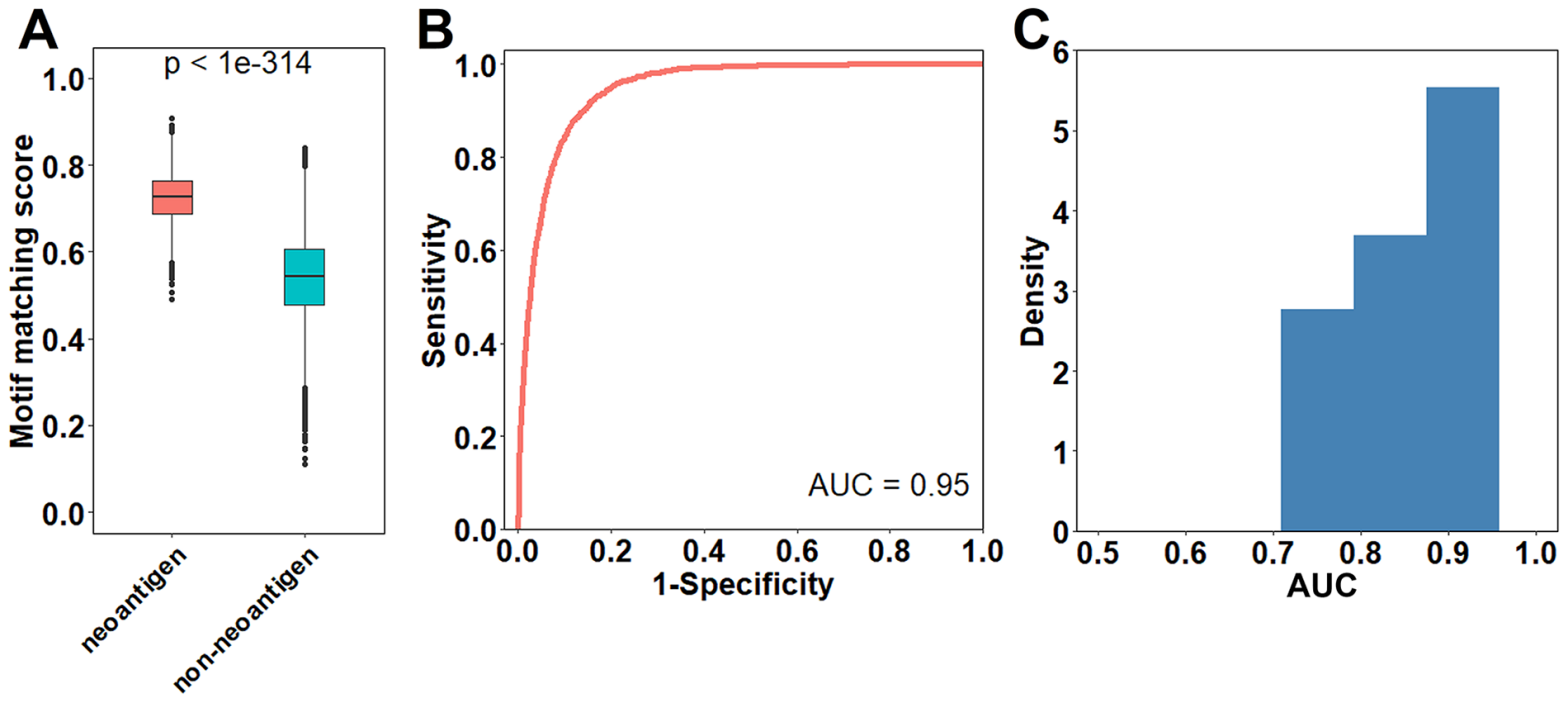
The performance of computed motifs in screening neoantigens in TCGA-READ datasets. (**A**) For common allele HLA-A02:01, the neoantigens show significantly higher motif matching scores compared to non-neoantigens. (**B**) Applying the computed motif to screen neoantigens for HLA-A02:01 resulted in an AUC of 0.95. (**C**) The computed motifs of 13 rare alleles have high performance in screen corresponding neoantigens. The distributions of AUCs are shown

We first focused on HLA-A02:01, the most frequent allele in TCGA READ datasets. We identified 2426 neoantigens for HLA-A02:01 among all 9-mer mutated peptides. As shown in Figure 6A, the neoantigens have significantly higher motif matching scores compared to non-neoantigens (*P* < 1e–314). Applying the motif matching scores to discriminate neoantigens versus non-neoantigens achieved an AUC of 0.95 (Fig. 6B). Using real motifs derived from experimental data, we observed similar results with an AUC of 0.95 (Supplementary Fig. S4A).

Among the 99 MHC I alleles in TCGA READ data, 19 alleles lack experimental data. We next evaluated the performance of motif matching scores on these 19 rare alleles. Similarly, we identified 9-mer neoantigens for each allele as previously described. Among the 19 alleles, 6 alleles were found to have no corresponding neoantigens. Applying the motif matching scores of the 13 remaining alleles to discriminate neoantigens from non-neoantigens resulted in AUCs ranging from 0.75 to 0.94 (Fig. 6C), with the mean AUC being 0.85.

To further validate the performance of our computed motifs in screening neoantigens, we applied NetMHCPan-4.1 as an orthogonal method to identify neoantigens and re-calculated the AUCs of motif matching scores. Consistently, we observed high AUCs of both computed (0.81–0.98 with mean 0.92, Supplementary Fig. S4B) and real motifs (0.73–0.98 with mean 0.94, Supplementary Fig. S4C) for common alleles. More importantly, for those rare alleles without experimental data, the computed motifs also achieved high AUCs ranging from 0.70 to 0.94, with a mean of 0.85 (Supplementary Fig. S4D).

In addition, we compared the speed of our motif matching method to that of the deep learning-based algorithm MHCflurry2.0. We randomly extracted 10 000 9-mer mutated peptides from TCGA-READ data and applied both methods to calculate motif-matching scores or presentation scores of each peptide for HLA-A02:01. Our motif matching method only took 0.381 s to finish calculation, while MHCflurry2.0 took 10.198 s and NetMHCPan-4.1 took 13.241 s to finish calculation. Considering 11672 single nucleotide variations of 69 patients in TCGA-READ cohort, each of them corresponds to (8 + 9 + …+ 15) potential candidate peptides, the number of peptides to be screened will be above 1 million. In this case, the motif matching method only requires about 38 s, while MHCflurry 2.0 requires 17 min for calculation. It should be noted that there are nearly 2 million cancer cases in the US in year 2020, screening neoantigens for these cases with motif matching method takes only 12 h, which is 26 times faster than MHCflurry2.0.

These results suggest that the generated motifs are effective at identifying neoantigens for both common and rare alleles. Also, in comparison with deep learning-based binding affinity predictors, calculating motif matching scores directly is much faster.

## 4 Conclusions

We presented a de novo generation framework PepPPO to characterize binding motifs for any given MHC Class I proteins via generating repertoires of binding peptides. We found that the characterized motifs from peptides generated by PepPPO are highly correlated with experimentally derived motifs and also highly robust. We applied the characterized binding motifs to screen neoantigens in rectum cancer, and demonstrated that motifs are effective in rapid screening of neoantigens for both common and rare human alleles. This sheds light on the development of rapid neoantigen screen techniques for precision therapy.

A limitation of PepPPO is that it employs the predictions from MHCflurry2.0 as the rewards to guide the optimization; if the predictions are highly uncertain, they could lead the agent to explore in the wrong direction. Therefore, incorporating the uncertainty of predictions into the framework can be a highly interesting and challenging future research direction. In addition, although peptide-MHC binding predictors (e.g. MHCflurry2.0) have been demonstrated with high accuracies, the characterized motifs for rare alleles need to be experimentally validated.

## Supplementary Material

btad055_Supplementary_Data
