# Supplementary material for: Binding peptide generation for MHC Class I proteins with deep reinforcement learning
Source: Bioinformatics. 2023 Jan 24;39(2):btad055. doi: 10.1093/bioinformatics/btad055 (PMC9907221; doi:10.1093/bioinformatics/btad055)
Supplement: btad055_Supplementary_Data [file btad055_supplementary_data.zip › suppl.pdf]

Supplementary Information for Binding Peptide Generation for MHC Class I Proteins with Deep Reinforcement Learning

S1 Implementation of Baseline methods

Algorithm S1 Monte Carlo Tree Search for Peptide Generation

Require: $r(.,m)$, c_{puct}, N, minLen, maxLen
$U = \{\}$
for $n = 1$ to N **do**
  ▷ root node of search tree T
  $p_0 =$ empty sequence; $\tau^n = \{\}$
  for $l = 1$ to maxLen **do**
    ▷ expansion
    for each amino acid o **do**
      $p'_l = p_{l-1} + o$
      if $p'_l \notin T$ **then**
        $W(p'_l) = 0$; $N(p'_l) = 0$; $P(p'_l) = 0$
        add p'_l as a child of p_{l-1} into T
      end if
    end for
    ▷ selection
    select a child p_l of p_{l-1} with the maximum value $a_k = \arg\max_a \frac{W(p_l)}{N(p_l)} + c_{puct} * \sqrt{\frac{2N(p_{l-1})}{1+N(p_l)}}$
    if $N(p_l) = 0$ and length(p_l) $\geq$ minLen **then**
      $P(p_l) = r(p_l, m)$
    end if
    $N(p_l) = N(p_l) + 1$
    add p_l into τ^n
    if $P(p_l) > \sigma$ **then**
      break
    end if
  end for
  ▷ backpropagation
  $r^n = 0$
  for each p_l along path τ^n from p_{maxLen} to p_0 **do**
    if $P(p_l) > r^n$ **then**
      $r^n = p_l.P$
    end if
    $W(p_l) = W(p_l) + r^n$
  end for
  ▷ output
  add p_l with maximum $P(p_l)$ into U
end for
return U

•`MCTS`: Monte Carlo Tree Search.

Given an MHC protein m, `MCTS` generates peptide sequences by adding one amino acid step by step until reaching the maximum length of 15 amino acids or with the presentation scores greater than a threshold. All the generated peptides with no less than 8 amino acids will be evaluated by `MHCflurry2.0`. The approach of `MCTS` is described in Algorithm S1. The hyper-parameter c_{puct} is set as 0.5.

•`BO-VAE`: Bayesian Optimization with the Variational Autoencoder (VAE).

In `BO-VAE`, a VAE model is pre-trained to convert peptide sequences of variable lengths into continuous latent embeddings of a fixed size with a single-layer LSTM. The reconstruction accuracy of peptide sequences with this VAE model is above 95%. Given an allele, the bayesian optimization algorithm with RBF kernel and the Upper Confidence Bound (UCB) is then employed to optimize random latent embeddings with maximum t steps so that the peptide sequences decoded from the optimized latent embeddings are with the high presentation scores. At each step, `BO-VAE` evaluates the presentation scores of the generated peptides with `MHCflurry2.0`, and stops the optimization as the peptides have the presentation scores greater than the threshold σ or it reaches the maximum step t. We implement the bayesian optimization algorithm using the BoTorch (Balandat *et al.*, 2020). We train the VAE model for 2e6 steps. We fine-tune the hyper-parameters and set the dimension of hidden layers for VAE as 64, the dimension of latent

embeddings as 8, the batch size as 256, the coefficient for KL loss as 0.001, the maximum steps t for bayesian optimization as 5, and the number of initial points as 500.

•`BP-VAE`: Back Propagation with the Variational Autoencoder.
In `BP-VAE`, a student model with the variational autoencoder same as that in the `BO-VAE` is employed to learn the presentation scores from `MHCflurry2.0`. After getting the trained student model, `BP-VAE` can generate peptides by optimizing the latent embeddings of peptides through gradient ascent to maximize the predicted presentation scores from the student model. The decoded peptide sequences with t steps will be evaluated by `MHCflurry2.0`. We fine-tune the VAE model in `BO-VAE` with the student model for 50,000 steps. We fine-tune the hyper-parameters and set the maximum step t as 50 and the learning rate of gradient ascent as 10.

•`sPWM`: Sampling from Position Weight Matrix.
For each MHC protein, `sPWM` generates peptides of length l by sampling from the amino acid distributions of all the l positions described in the Section 2.4.2. To decide the length l of the generated peptides, we sample length 8 with the probability 15%, length 9-11 with 20%, length 12 with 10%, and length 13-15 with 5%, respectively. The probabilities are determined according to that most qualified peptides have length from 8 to 11 (Trolle *et al.*, 2016).

•`Random`: Randomly generating peptide sequences of length from 8 to 15. The length of each peptide is determined in the same way as in `sPWM`.

S2 Parameter Setup

Table S1. Selected MHC I Proteins for Benchmark

Common Alleles			Rare Alleles		
HLA-A	HLA-B	HLA-C	HLA-A	HLA-B	HLA-C
HLA-A*02:01	HLA-B*27:05	HLA-C*12:02	HLA-A*25:56	HLA-B*54:38	HLA-C*06:147
HLA-A*03:01	HLA-B*07:02	HLA-C*05:01	HLA-A*25:54	HLA-B*54:33	HLA-C*07:68
HLA-A*11:01	HLA-B*57:01	HLA-C*16:01	HLA-A*25:50	HLA-B*56:14	HLA-C*07:302
HLA-A*01:01	HLA-B*40:02	HLA-C*08:02	HLA-A*25:36	HLA-B*54:06	HLA-C*07:242
HLA-A*24:02	HLA-B*15:01	HLA-C*15:02	HLA-A*25:18	HLA-B*51:261	HLA-C*12:183
HLA-A*68:01	HLA-B*08:01	HLA-C*06:02	HLA-A*02:432	HLA-B*15:180	HLA-C*07:549
HLA-A*68:02	HLA-B*40:01	HLA-C*07:01	HLA-A*32:78	HLA-B*59:06	HLA-C*07:518
HLA-A*29:02	HLA-B*58:01	HLA-C*03:03	HLA-A*26:94	HLA-B*58:07	HLA-C*07:475
HLA-A*02:03	HLA-B*44:02	HLA-C*03:04	HLA-A*26:34	HLA-B*56:08	HLA-C*07:401
HLA-A*31:01	HLA-B*51:01	HLA-C*14:02	HLA-A*26:16	HLA-B*56:03	HLA-C*07:291

We listed the hyper-parameters of the peptide mutation environment, the policy network and the RL agent in Table S2. We selected an optimal set of hyperparameters including the discount factor, the entropy coefficient and the maximum steps by a grid search. We found little improvement when increasing the dimension or number of layers of policy network and value network. In the implementation of `PepPPO`, for each iteration, we ran 30 environments in parallel for 128 timesteps and collect trajectories with 3,840 timesteps in total. We trained `PepPPO` for 1e7 timesteps (i.e., 2605 iterations). We set all the other hyper-parameters for the RL agent as the default hyper-parameters provided by the stable-baselines3 (Raffin *et al.*, 2019), as we find little performance improvement from tuning these parameters. We initialized the state s_0 with a randomly selected pseudo sequence of MHC protein and a random peptide. When randomly initializing the peptide, the length of the peptide is determined in the same way as in `sPWM`. We pre-trained the `PepPPO` with the expert policy for 5,000 steps in total. During the pre-training, at each step, we randomly generated 128 states using the above strategy for initialization, and then train the policy using the corresponding actions from the expert policy π_{ept}. In addition to leveraging the expert policy through the pre-training, we also used the expert policy π_{ept} to generate actions with 80% probability at the beginning of training to guide the exploration of our policy. We decreased the percentage of actions from the expert policy by 5% every 40,000 steps. In the main manuscript, `PepPPO` refers to the one with the pre-training by default, instead of the one with the actions from expert policy. For the `PepPPO` with the diversity-promoting buffer, we set the maximum buffer size as 20,000, and define the actions with frequencies greater than 0.02 as the frequent actions; otherwise, the infrequent actions. We set the threshold of presentation score as 0.75. We demonstrate that 0.75 is a proper threshold for presentation score, as we observe that it ranks at top 2.6% in the MULTIALLELIC dataset (i.e., a dataset used in O'Donnell *et al.* (2020)) and leads to only 1.9% at false positive rate (FPR) and 65.8% at true positive rate (TPR) on the MULTIALLELIC-RECENT dataset. We empirically found that 0.75 can make a good tradeoff between FPR and TPR, as a lower cutoff can lead to more false positives (e.g., 5.1% at FPR for cutoff 0.5) and a higher cutoff can lead to less true positives (e.g., 45.0% at TPR for cutoff 0.9).

S3 Discussion on Reward Model

In `PepPPO`, we used the peptide-MHC binding predictions from `MHCflurry2.0` as the rewards due to the lack of experimentally measured binding affinities for all the peptide-MHC pairs. `MHCflurry2.0` which consists of two modules - binding affinity predictor (BA) and antigen processing predictor, is one of the state-of-the-art peptide-MHC interaction predictor. Specifically, BA achieved good performance for most alleles (i.e., 214 of 236 alleles, 91%) having AUCs of at least 0.90; and `MHCflurry2.0` used in `PepPPO` even outperformed BA with 51% improvement in terms of positive predictive value. The superior performance of `MHCflurry2.0` demonstrates that `MHCflurry2.0` can provide `PepPPO` with reliable rewards and enable it to generate reliable positive peptides for the characterization of high-quality binding motifs. In addition to the high accuracy, `MHCflurry2.0` provides well-organized

Table S2. Experimental Setup for `PepPPO`

description	value
maximum steps T	8
presentation score threshold σ	0.75
discount factor γ	0.9
GAE parameter λ	0.95
hidden dimension of W_2^m	128
latent dimension of $\overrightarrow{\mathbf{h}}_i/\overleftarrow{\mathbf{h}}_i/\mathbf{h}^m$	48
hidden dimension of policy & value networks	40
hidden layers of policy & value networks	2
number of steps per iteration	3,840
entropy coefficient	0.01
batch size for policy update	64
number of optimization epochs per iteration	10
clip range	0.2
learning rate	3e-4

source code in Python and detailed documentation, which allows us to integrate the predictor easily into our framework; in contrast, other methods such as `NetMHCPan-4.1` only provide executable programs without source code and limited documentation. Considering all these factors, we adopted `MHCflurry2.0` as the reward function in `PepPPO`.

S4 Algorithm of `PepPPO`

Algorithm S2 describes the learning algorithm of `PepPPO`. Given the pre-trained `MHCflurry2.0` function $f(r|m,p)$ as the reward function in the environment and the expert policy π_{ept}, we first pretrain the policy network $\pi_\theta(a|s)$ using $\pi_{ept}(a|s)$for N_{pre} steps, and then train the policy network $\pi_\theta(a|s)$ and value network $V_\theta(s)$ by interacting with the environment.

Algorithm S2 Learning Algorithm of `PepPPO` with Pre-training and Diversity-promoting Buffer

Require: $f(r|m,p)$, $\pi_{ept}(a|s)$, N_{pre}, maxStep, iterStep, epoch

initialize diversity-promoting buffer $B = \emptyset$
▷ pretrain $\pi_\theta(a|s)$ using $\pi_{ept}(a|s)$
for $n = 1$ to N_{pre} **do**
  sample state-action pairs $\{(a,s)\}$ with $\pi_{ept}(a|s)$
  update $\pi_\theta(a|s)$ using loss L_{pre} over $\{(a,s)\}$ (Equation 10)
end for
▷ train $\pi_\theta(a|s)$ by interacting with the environment
t = 0
while $t <$maxStep **do**
  collect trajectories $\{(s_0, a_0, r_0, ..., s_T, a_T, r_T)\}$ by modifying peptides with $\pi_\theta(a|s)$ and receiving rewards $\{r_t\}$ from $f(r|m,p)$
  add trajectories $\{(s_0, a_0, ..., s_T, a_T)\}$ with s_T including qualified peptides into B
  calculate returns $\{(\hat{R}_0, \hat{R}_1, ...\hat{R}_T)\}$ for all states in trajectories using $\{r_t\}$
  calculate advantages $\{(\hat{A}_0, \hat{A}_1, ..., \hat{A}_T)\}$ using $\{r_t\}$ and $V_\theta(s)$ (Equation 6)
  for $n = 1$ to epoch **do**
    sample $\{(s_t, a_t, \hat{A}_t, \hat{R}_t)\}$ from collected data
    calculate $L^{CLIP}(\theta)$ and $L^V(\theta)$ over $\{(s_t, a_t, \hat{A}_t), \hat{R}_t)\}$ (Equation 5 and 8)
    sample $\{(s_t, a_t)\}$ from B
    calculate $L^B(\theta)$ over $\{(s_t, a_t)\}$ (Equation 11)
    calculate final objective $L(\theta)$ using $L^{CLIP}(\theta)$, $L^V(\theta)$, $L^B(\theta)$ and entropy $H(\theta)$ (Equation 12)
    update parameters θ of $\pi_\theta(a|s)$ and $V_\theta(s)$ using loss $L(\theta)$
  end for
  $t = t +$ iterStep
end while

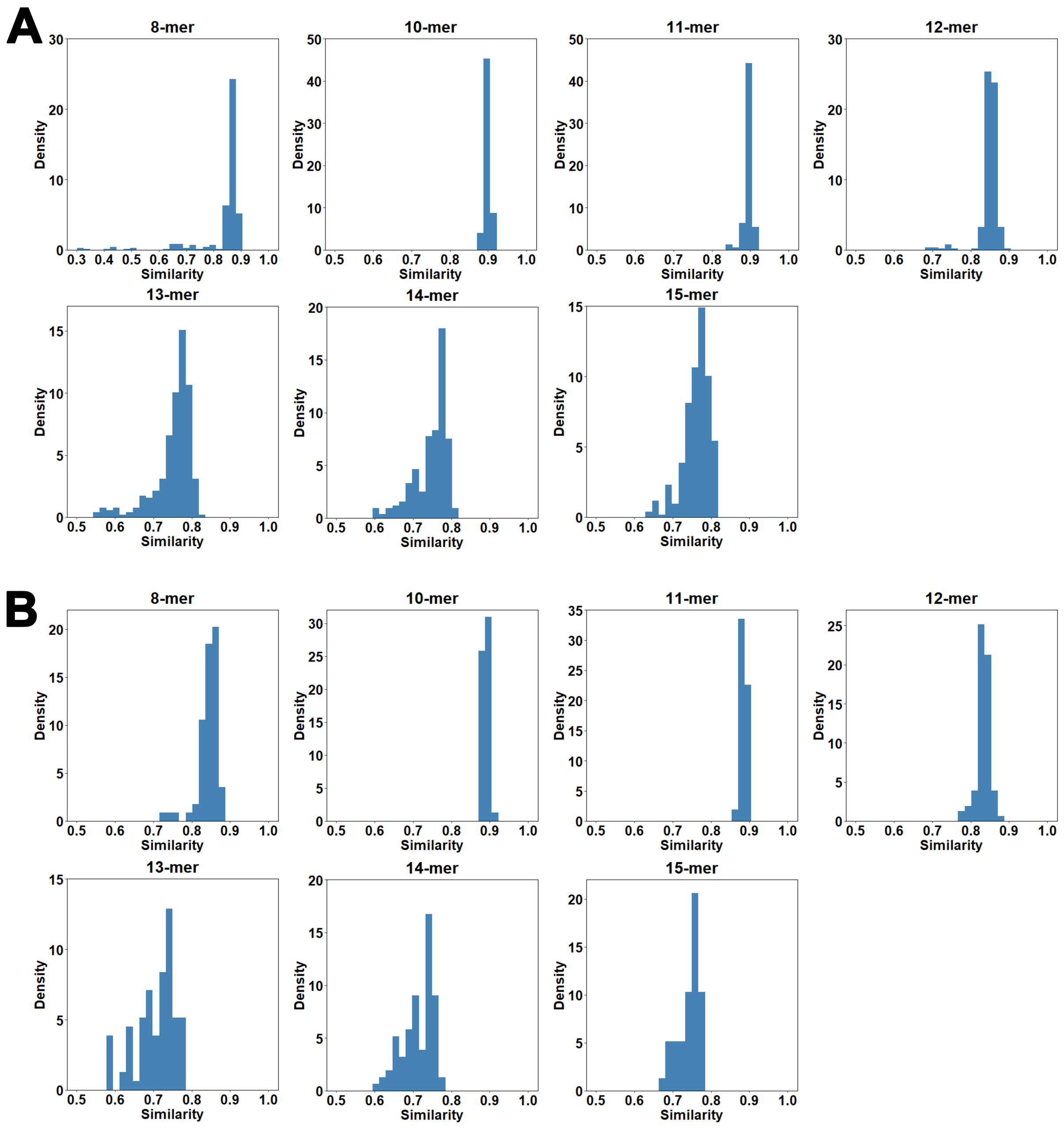

Fig. S1: The similarity between 8∼15-mer motifs across five runs of PepPPO for 30 common (A) and 30 rare (B) alleles.

S5 Supplementary Figures

References

Balandat, M., Karrer, B., Jiang, D. R., Daulton, S., Letham, B., Wilson, A. G., and Bakshy, E. (2020). BoTorch: A Framework for Efficient Monte-Carlo Bayesian Optimization. In *Advances in Neural Information Processing Systems 33*.

O'Donnell, T. J., Rubinsteyn, A., and Laserson, U. (2020). Mhcflurry 2.0: Improved pan-allele prediction of mhc class i-presented peptides by incorporating antigen processing. *Cell Systems*, **11**(1), 42–48.e7.

Raffin, A., Hill, A., Ernestus, M., Gleave, A., Kanervisto, A., and Dormann, N. (2019). Stable baselines3. https://github.com/DLR-RM/stable-baselines3.

Trolle, T., McMurtrey, C. P., Sidney, J., Bardet, W., Osborn, S. C., Kaever, T., Sette, A., Hildebrand, W. H., Nielsen, M., and Peters, B. (2016). The length distribution of class i–restricted t cell epitopes is determined by both peptide supply and MHC allele–specific binding preference. *The Journal of Immunology*, **196**(4), 1480–1487.

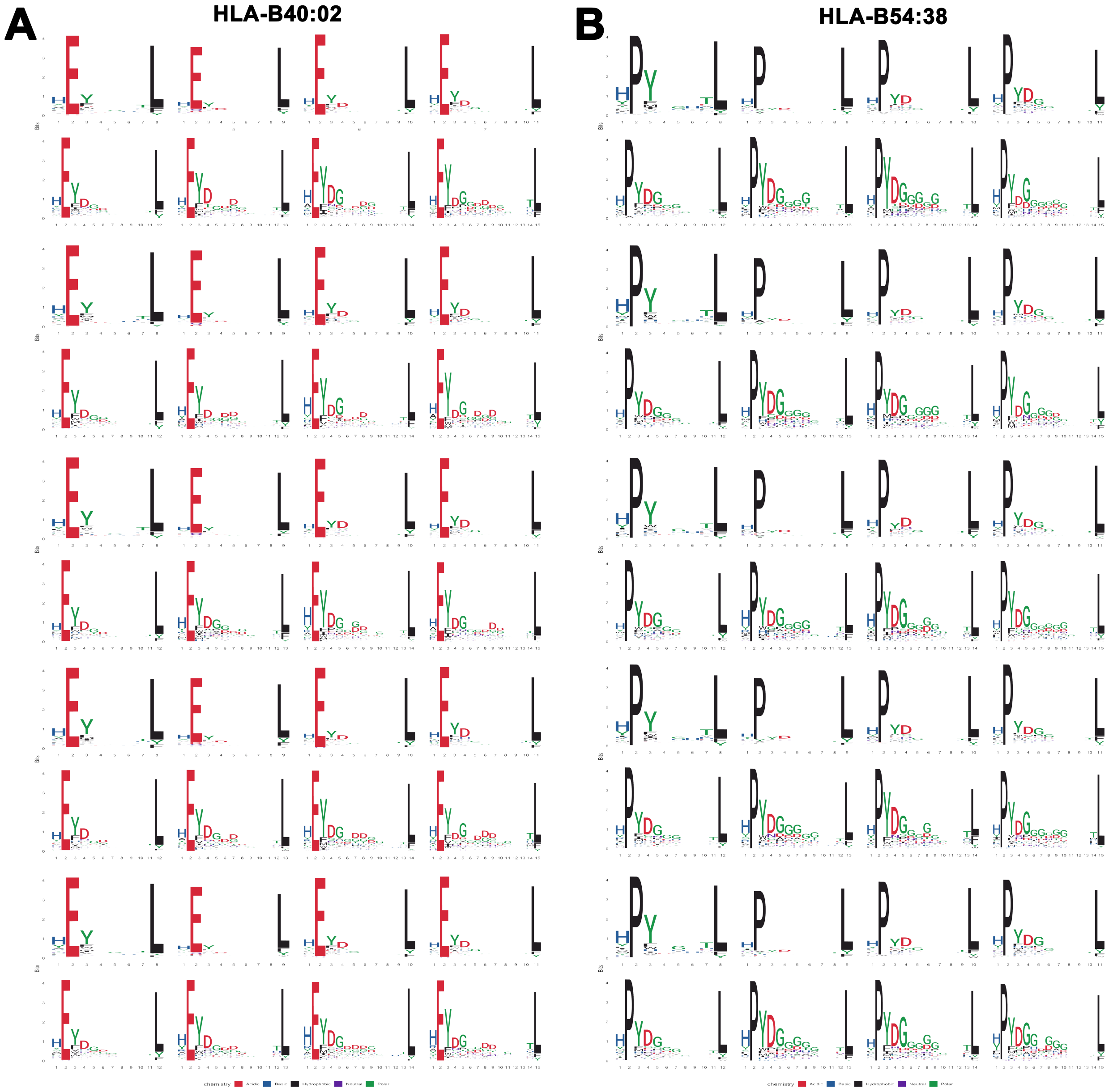

Fig. S2: Motifs derived from PepPPO generated peptides are highly consistent with random initiations. We select 1 common allele HLA-B40:02 and one rare allele HLA-B54:38 to visualize motifs derived from 5 runs of PepPPO.

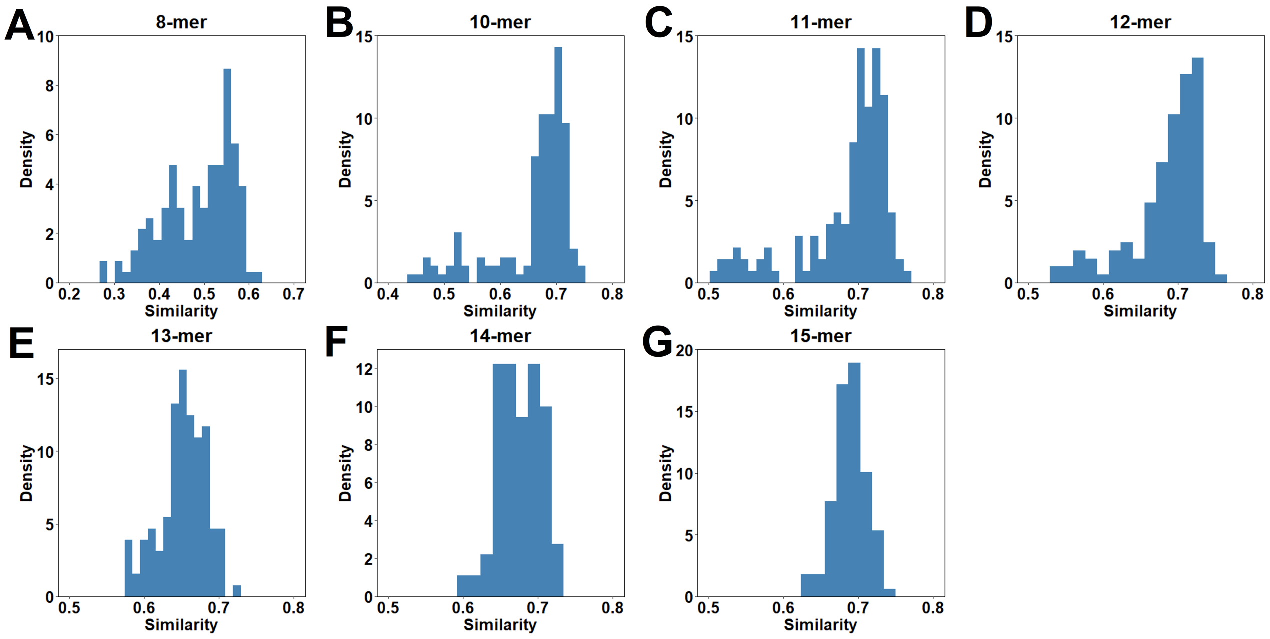

Fig. S3: Motifs derived from PepPPO generated peptides are highly correlated with the real motifs derived from experimental data. The similarity between 8 to 15-mer motifs are shown.

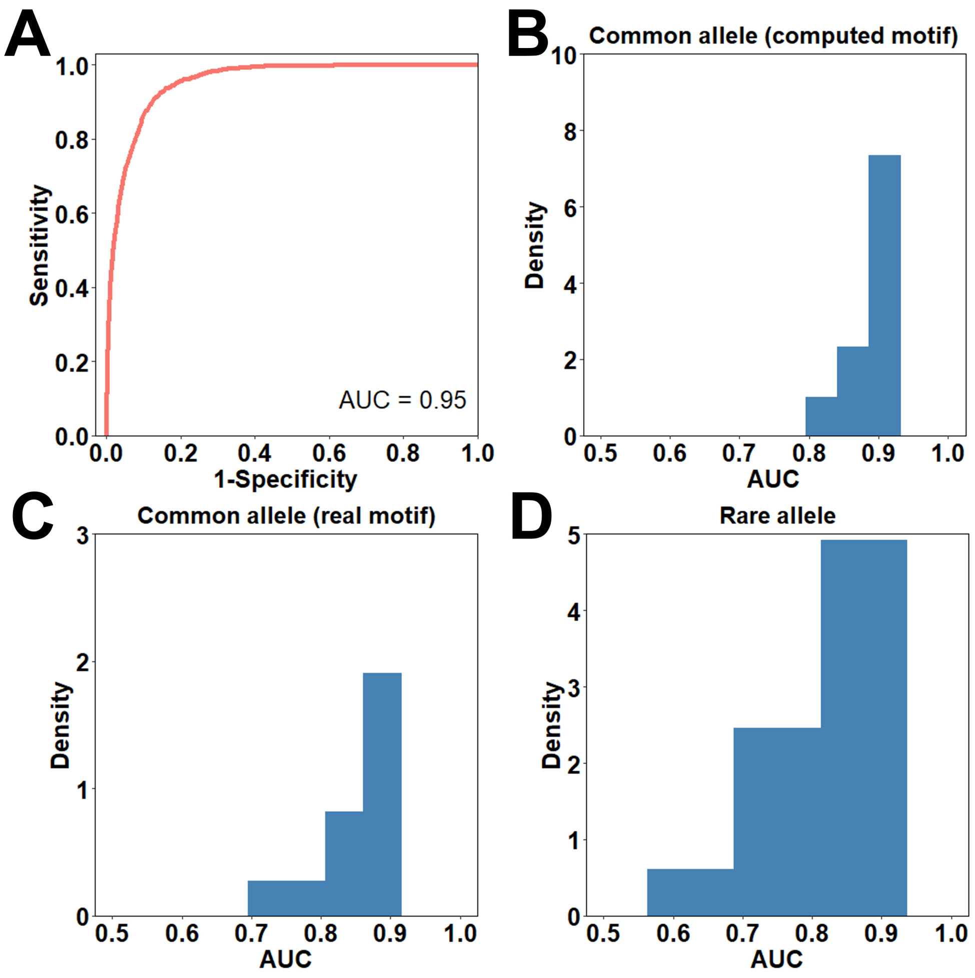

Fig. S4: The AUC of motifs derived from the experimental data in identify neoantigens for HLA-A02:01 (A); the distribution of AUCs for computed motifs (B) and real motifs (C) on common alleles with neoantigens labeled by `NetMHCPan-4.1`; the distribution of AUCs for computed motifs on rare alleles with neoantigens labeled by `NetMHCPan-4.1` (D).
